# The Influence of Age on Interaction between Breath-Holding Test and Single-Breath Carbon Dioxide Test

**DOI:** 10.1155/2017/1010289

**Published:** 2017-01-31

**Authors:** Nikita Trembach, Igor Zabolotskikh

**Affiliations:** Kuban State Medical University, Sedin Str. 4, Krasnodar 350012, Russia

## Abstract

*Introduction*. The aim of the study was to compare the breath-holding test and single-breath carbon dioxide test in evaluation of the peripheral chemoreflex sensitivity to carbon dioxide in healthy subjects of different age.* Methods*. The study involved 47 healthy volunteers between ages of 25 and 85 years. All participants were divided into 4 groups according to age: 25 to 44 years (*n* = 14), 45 to 60 years (*n* = 13), 60 to 75 years (*n* = 12), and older than 75 years (*n* = 8). Breath-holding test was performed in the morning before breakfast. The single-breath carbon dioxide (SB-CO_2_) test was performed the following day.* Results*. No correlation was found between age and duration of breath-holding (*r* = 0.13) and between age and peripheral chemoreflex sensitivity to CO_2_ (*r* = 0.07). In all age groups there were no significant differences in the mean values from the breath-holding test and peripheral chemoreflex sensitivity tests. In all groups there was a strong significant inverse correlation between breath-holding test and SB-CO_2_ test.* Conclusion*. A breath-holding test reflects the sensitivity of the peripheral chemoreflex to carbon dioxide in healthy elderly humans. Increasing age alone does not alter the peripheral ventilatory response to hypercapnia.

## 1. Introduction

The role of peripheral chemoreflex sensitivity to hypoxia and hypercapnia in the pathogenesis of various pathological conditions has garnered much attention in recent years. The degree of impairment in cardiorespiratory system reflex regulation is a marker of disease progression and its prognosis [1, 2]. Increased peripheral chemoreflex sensitivity is associated with a decrease in arterial baroreflex sensitivity in chronic cardiovascular diseases [3], which is a risk factor for hemodynamic instability.

The study of the peripheral chemoreflex sensitivity is traditionally performed by a hypoxic test [4–6]; however persistent hypoxia occurs during these techniques, which can potentially lead to respiratory depression due to central effects [5]. Furthermore, there is a potential risk of adverse incidents related to hypoxia, especially in high risk patients. The method of single-breath carbon dioxide test, designed by McClean et al. [7], is an alternative method of evaluating peripheral chemoreflex sensitivity and is relatively safe compared with hypoxic tests. In addition, it has worked well in clinical practice [8]. However, this method also requires sophisticated equipment, which limits its application in routine practice.

The duration of a voluntary apnea depends on several factors, and one of them is the sensitivity of the peripheral chemoreflex [9]. However, the properties of the respiratory system may change with the age and respiratory biomechanics in the elderly may be different from that in young adults. Data on the effect of age on the peripheral chemoreflex sensitivity are controversial; there are works showing an increase [10] or a decrease [11] of sensitivity. Other researchers found no effect of age on peripheral chemoreflex [12]. However, much of the research describes the hypoxic test using hypoxic gas mixture (pure nitrogen) to reduce the arterial oxygen saturation to 65–85%. Currently there is little data on the effect of age on the sensitivity of peripheral chemoreceptors to carbon dioxide.

The aim of the study was to assess whether the breath-holding test reflects peripheral chemoreflex sensitivity to carbon dioxide in healthy subjects of different ages.

## 2. Methods

The study involved 47 healthy volunteers between the ages of 25 and 85 years (23 males, 24 females). The study was approved by the local ethics committee. All subjects provided signed informed consent to both tests. Volunteers were recruited from the population during 2015-2016 years. All participants were divided into 4 groups according to age: 25 to 44 years (*n* = 14), from 45 to 60 years (*n* = 13), from 60 to 75 years (*n* = 12), and more than 75 years (*n* = 8). No subjects had a history of chronic respiratory or cardiovascular disease, alcohol abuse, or smoking. Before the study, all patients were weighed, the body mass index was calculated, and respiratory function was evaluated using spirometry (Table 1).

In all participants, the breath-holding test was performed in the morning before breakfast. The single-breath carbon dioxide test was performed the following day.

The single-breath carbon dioxide test was performed as follows. The participant's nose was clamped using a soft grip. Breathing through the mouth was monitored using a mouthpiece connected to a pneumatic respiratory valve separating the inhaled gas mixture from exhaled air. The inspiratory port was connected to a T-shaped valve in such a way that ventilation is carried out from either a rubber bag or a 2 L tank, which was filled after each inhalation of the gas mixture containing 13% CO_2_ or atmospheric air. After a brief period of eupnoea (approximately 5 min), in the expiratory phase, the T-shaped valve was switched to breathing a mixture with high CO_2_ content so that the next breath was taken using this mixture. The valve was then switched to atmospheric air. On average, 10 breaths of the hypercapnic mixture were taken with intervals of 2 min of breathing room air. Respiratory rate and tidal volume were estimated breath to breath with the calculation of minute ventilation (Volumeter Blease, United Kingdom). The CO_2_ fraction in the exhaled mixture was measured using a sidestream gas analyser (Nihon Kohden, Japan). The average minute ventilation was calculated from the data of the last five breaths before breathing the hypercapnic mixture as the control. Likewise, the average FetCO_2_ was determined during these breaths and used as the control FetCO_2_. The ventilation response to a hypercapnic stimulus was determined as the average of the two highest rates of MV (during the first 20 seconds after the stimulus, breaths beyond this time were excluded to minimize the contribution of central chemoreception). Poststimulus FetCO_2_ was also assessed during these cycles. The ventilation response to breathing a hypercapnic mixture was calculated by the formula: (poststimulus  MV − control  MV)/((poststimulus  FetCO_2_ − control  FetCO_2_)×(*P*_atm_ − 47)), where *P*_atm_ represents the atmospheric pressure in mmHg and 47 is the saturated water vapour pressure in mmHg. The median of all 10 episodes was taken as the sensitivity of the peripheral chemoreflex, expressed in L/min/mmHg.

The breath-holding test was performed as follows: voluntary breath-holding duration was assessed three times, with 10 min intervals of normal resting breathing. After inspiration of an atmospheric air volume equal to 2/3 of the vital lung capacity ±15%, the participant was asked to hold their breath and the duration of voluntary apnea was measured from the beginning of the voluntary inspiration until reflex contractions of the diaphragm were noted by palpation. A mean value of the duration of the three samples was calculated.

Data are presented as mean ± standard deviation due to normal distribution (Shapiro–Wilk test). To assess the relationship between the two methods, Pearson's correlation coefficient was calculated.

## 3. Results of the Study

**Figure 1:**
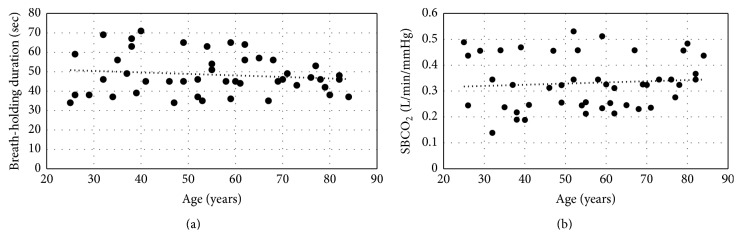
The relationship of age and breath-holding duration (a) and age and peripheral chemoreflex sensitivity to carbon dioxide (b).

In total the average sensitivity of peripheral chemoreflex was 0.326 ± 0.107 L/min/mm Hg; the average duration of breath-holding test was 49 ± 10 seconds. There was a positive correlation between the subjects' height and peripheral chemoreflex sensitivity (*r* = 0.45,  *R*^2^ = 0.2, and *p* < 0.05); no correlation was found between chemoreflex sensitivity and other characteristics. We also found a positive correlation between the duration of breath-holding and vital lung capacity (*r* = 0.54,  *R*^2^ = 0.29, and  *p* < 0.05). No correlation was found between age and breath-holding duration (*r* = 0.13,  *R*^2^ = 0.2) (Figure 1(a)) and between age and peripheral chemoreflex sensitivity (*r* = 0.07,  *R*^2^ = 0.2) (Figure 1(b)).

**Figure 2:**
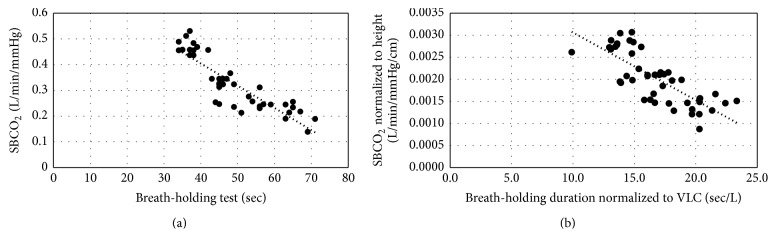
The relationship of breath-holding duration and peripheral chemoreflex sensitivity to carbon dioxide (SB-CO_2_) (a), breath-holding duration normalized to vital lung capacity (VLC), and peripheral chemoreflex sensitivity to carbon dioxide normalized to height (b).

In general, we found a significant inverse correlation between the results of the two tests (*r* = −0.88,  *R*^2^ = 0.78, and  *p* < 0.001) (Figure 2(a)). The linear correlation equation for the relationship was *y* = −0.00863 × *x* + 0.75. Also, a significant inverse correlation was found between breath-holding duration normalized to vital lung capacity and peripheral chemoreflex sensitivity, normalized to subjects' height (*r* = −0.8,  *R*^2^ = 0.65, and *p* < 0.001) (Figure 2(a)). The linear correlation equation for this relationship was *y* = −0.000158 × *x* + 0.00465.

In all age groups there were no significant differences in the mean values of the breath-holding duration and peripheral chemoreflex sensitivity to carbon dioxide (Table 2). In all groups there was a strong significant inverse correlation between breath-holding duration and peripheral chemoreflex sensitivity.

## 4. Discussion

The duration of breath-holding after deep inspiration is a function of several factors [13]: chemoreception, mechanoreception (receptors of light stretching), the impact of descending cortical respiratory drive, and a cognitive component, of which the first two are involuntary, but the most important components [14]. The duration of voluntary apnea doubled after breathing a hyperoxic mixture or after prehyperventilation [15]. On the other hand, the breath-holding duration was reduced under hypoxemic and hypercapnic conditions [16, 17]. Thus, it is not surprising that the duration of breath-holding had a strong inverse correlation with the SB-CO_2_ test. Davidson et al. [18] reported higher *P*_ET_CO_2_ values after breath-holding in subjects with prior carotid body resections for asthma compared to healthy volunteers, which suggests great contributing of peripheral chemoreception. Feiner et al. [19] showed that the peripheral chemoreception, but not central, makes the largest contribution to the breath-holding duration, but the peripheral ventilatory response to hypercapnia was not evaluated. Our work demonstrates the contribution of peripheral sensitivity to carbon dioxide to the breath-hold duration.

Importantly, we noted that increasing age has no effect on this pattern. The duration of breath-holding did not depend on the age and did not differ between the groups, although there is evidence that potential changes in the respiratory system and respiratory biomechanics associated with aging [20]. Structural changes of the intercostal muscles and joints and edge-vertebral joints may accompany the aging process, but these changes may not necessarily have been presented [21]. A reduction in the elastic properties of lung tissue also occurs with age [21], but this is often the result of comorbidity. The analysis of our results showed that the initial values of FEV_1_, vital capacity, tidal volume, and respiratory rate did not differ between age groups, indicating respiratory biomechanics likely were not markedly altered with age in our study. This observation could explain the absence of differences in the duration of breath-holding after a deep inspiration.

Existing works on the effect of age on the sensitivity of the peripheral chemoreflex represent conflicting results, from no change in sensitivity in the elderly [12] to an increase [10] or decrease [11]. However, most researchers used a hypoxic test and their works had a different design (steady-state, progressive, or transient methods) and included different age groups. It should be noted that unlike the trend for PaO_2_ to decrease with age the exchange of carbon dioxide varies with age much less with relatively unchanged PaCO_2_ [22]. The stability of PaCO_2_ with aging may have caused a lack of influence of age on the duration of breath-holding and on respiratory response to carbon dioxide. Martinez showed a decreased peripheral response to hypercapnia in elderly men (55 to 76 years old) compared to young men (25 to 38 years old), but there were some limitations due to a small sample size [23]. Our findings indicate that there is no relationship of age and sensitivity of peripheral chemoreceptors to carbon dioxide. In our study the average sensitivity of peripheral chemoreflex was 0.326 ± 0.107 L/min/mm Hg with no influence of age, so our data correlates with results of other authors, who described values of 0.34 ± 0.12 L/min/mm Hg [8] and 0.28 ± 0.04 L/min/mm Hg [24] in healthy subjects.

However, an increase in peripheral chemoreflex sensitivity to carbon dioxide may advance aging, because aging is often associated with concomitant diseases, resulting, as is known, in a change in the reflex regulation of the cardiorespiratory system and increase peripheral chemoreflex sensitivity [25]. Such diseases include chronic heart failure [1, 26], hypertension, chronic obstructive pulmonary disease, obstructive sleep apnea, and other conditions [27]. Thus, our findings support the thesis that the biological age does not always equate with chronological age and in the absence of chronic disease the peripheral chemoreflex remains intact.

Thus, a pattern marked by us in healthy people of different ages may be slightly different in situations that affect these factors (obesity, cardiac disease, respiratory diseases, etc.), and this fact requires further research.

The correlation with SB-CO_2_ test results and subjects' height observed in our study is consistent with those obtained by Chua and Coats [24], who also found similar relationship, but it was not statistically significant. Perhaps this is due to the fact that the number of observations in our work was greater, which could influence the statistics. The results indicate a positive correlation between the duration of breath-holding and vital lung capacity. The duration of voluntary apnea also depends on the lung volumes [28]. Previous studies showed that lung volumes greatly influence a breath-holding [29] and forced vital capacity was identified as a significant predictors of breath-hold duration [19].

## 5. Conclusion

A breath-holding test reflects the sensitivity of the peripheral chemoreflex to carbon dioxide in the healthy elderly. Increasing age alone does not alter the peripheral ventilatory response to hypercapnia.

## Figures and Tables

**Table 1:** Characteristics of the subjects.

	Age group			
	25–44 years	45–59 years	60–74 years	≥75 years
Average age, years	34 ± 5	53 ± 4	66 ± 4	79 ± 3
Weight, kg	72 ± 4	76 ± 4	74 ± 4	69 ± 4
Height, cm	167 ± 4	164 ± 5	165 ± 6	164 ± 4
FEV_1_ (% predicted)	98 ± 4	96 ± 6	97 ± 7	95 ± 5
VLC (% predicted)	101 ± 3	99 ± 4	95 ± 6	94 ± 6

FEV_1_: forced expiratory volume; VLC: vital lung capacity. Data are presented as mean ± standard deviation.

**Table 2:** Correlation between breath-holding duration and peripheral chemoreflex sensitivity to carbon dioxide in different age groups.

	Age group			
	25–44 years	45–59 years	60–74 years	≥75 years
Breath-holding duration, sec	51 ± 13	48 ± 11	48 ± 8	47 ± 7
Peripheral chemoreflex CO_2_ sensitivity, L/min/mmHg	0.317 ± 0.119	0.345 ± 0.105	0.312 ± 0.064	0.333 ± 0.124
Correlation coefficient	−0.93^*∗*^	−0.86^*∗*^	−0.83^*∗*^	−0.88^*∗*^

^*∗*^ *p* < 0.05.

## Abbreviations

SB-CO_2_: Single-breath carbon dioxide
FEV_1_: Forced expiratory volume
VLC: Vital lung capacity.
